# Supplementary material for: The USH3A causative gene clarin1 functions in Müller glia to maintain retinal photoreceptors
Source: bioRxiv. 2024 Mar 1:2024.02.29.582878. Preprint. [Version 1] doi: 10.1101/2024.02.29.582878 (PMC10925332; doi:10.1101/2024.02.29.582878)
Supplement: Supplement 1 [file NIHPP2024.02.29.582878v1-supplement-1.pdf]

bioRxiv preprint doi: https://doi.org/10.1101/2024.02.29.582878; this version posted March 1, 2024. The copyright holder for this preprint (which was not certified by peer review) is the author/funder, who has granted bioRxiv a license to display the preprint in perpetuity. It is made available under aCC-BY 4.0 International license.

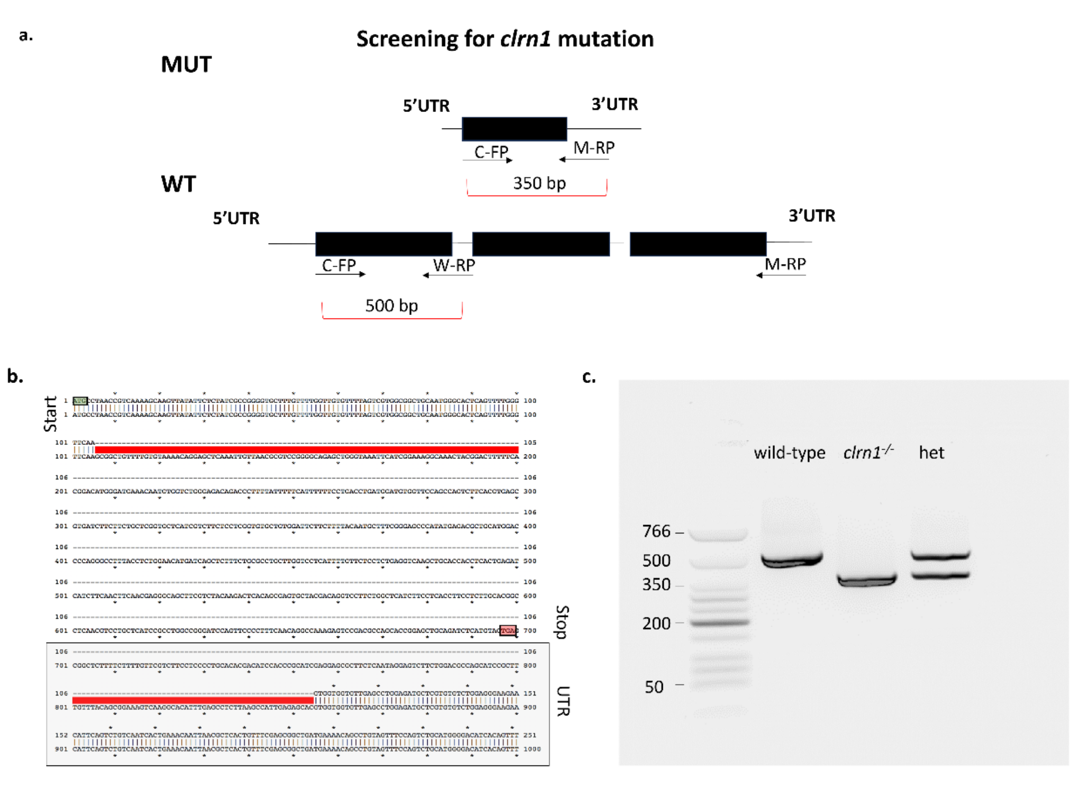

Supplemental Figure 1: Method to genotype for *clrn1*$^{-/-}$, wild-type, and heterozygous zebrafish

(a) Depiction of primer design for genotyping wild-type, heterozygous, and *clrn1*$^{-/-}$ zebrafish. A common forward primer (C-FP) was designed upstream of the exon 1 cut site, while a mutant reverse primer (M-RP) was designed downstream of the 3' UTR cut site, and W-RP was designed internal to the exon 1 cut site. (b) Generation of the large deletion in the *clrn1* coding sequence was confirmed with Sanger sequencing. (c) Example gel depicting the PCR amplification products for wild-type, *clrn1*$^{-/-}$, and heterozygous (het) zebrafish.

bioRxiv preprint doi: https://doi.org/10.1101/2024.02.29.582878; this version posted March 1, 2024. The copyright holder for this preprint (which was not certified by peer review) is the author/funder, who has granted bioRxiv a license to display the preprint in perpetuity. It is made available under aCC-BY 4.0 International license.

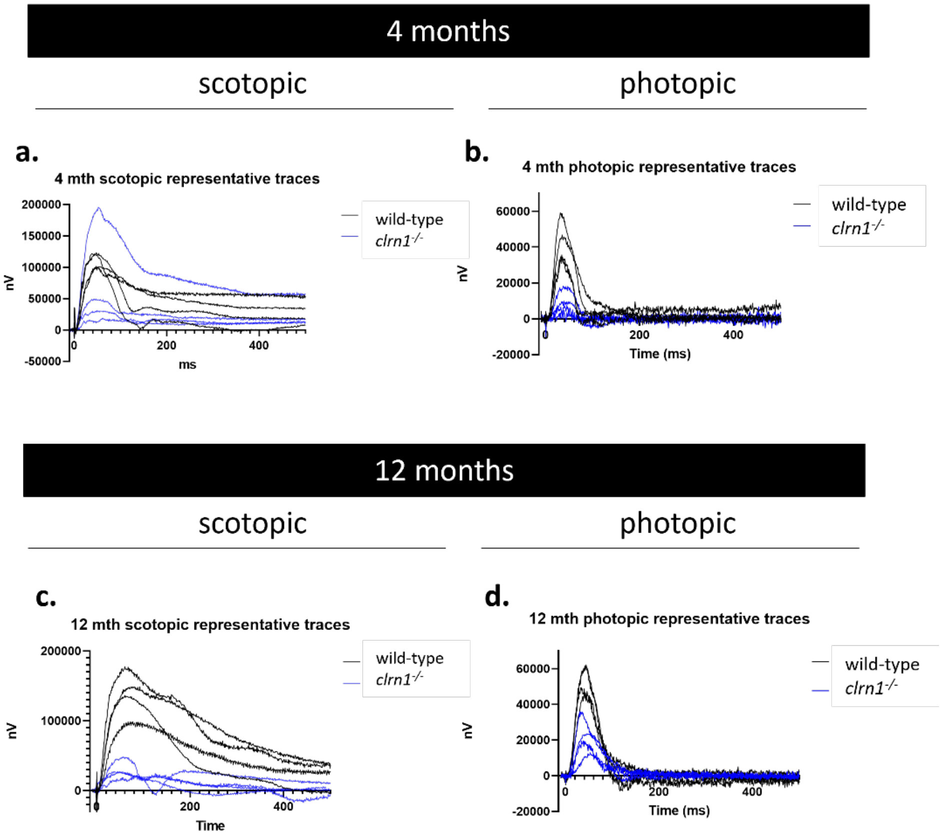

Supplemental Figure 2: Representative traces of photopic and scotopic responses. Representative traces from four 4 mpf wild-type and *clrn1*$^{-/-}$ scotopic (a) and photopic (b) responses to highlight high variability in the *clrn1*$^{-/-}$ scotopic b-wave. Representative traces from 12 mpf wild-type and *clrn1*$^{-/-}$ scotopic (c) and photopic (d) responses.

bioRxiv preprint doi: https://doi.org/10.1101/2024.02.29.582878; this version posted March 1, 2024. The copyright holder for this preprint (which was not certified by peer review) is the author/funder, who has granted bioRxiv a license to display the preprint in perpetuity. It is made available under aCC-BY 4.0 International license.

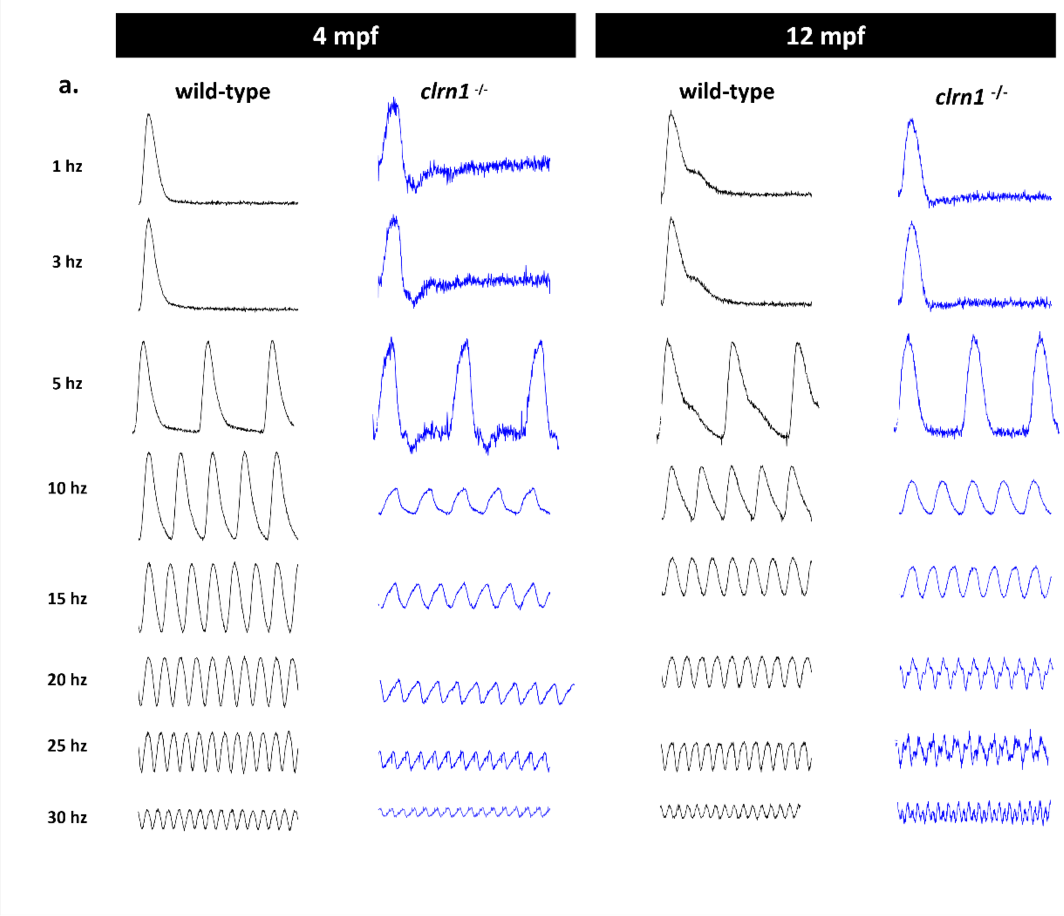

Supplemental Figure 3 Photopic flicker response are reduced in 4 and 12 mpf *clrn1$^{-/-}$* zebrafish

(a). Representative photopic flicker traces of 4 and 12 mpf wild-type and *clrn1$^{-/-}$* at a range of 1-30 hz.

bioRxiv preprint doi: https://doi.org/10.1101/2024.02.29.582878; this version posted March 1, 2024. The copyright holder for this preprint (which was not certified by peer review) is the author/funder, who has granted bioRxiv a license to display the preprint in perpetuity. It is made available under aCC-BY 4.0 International license.

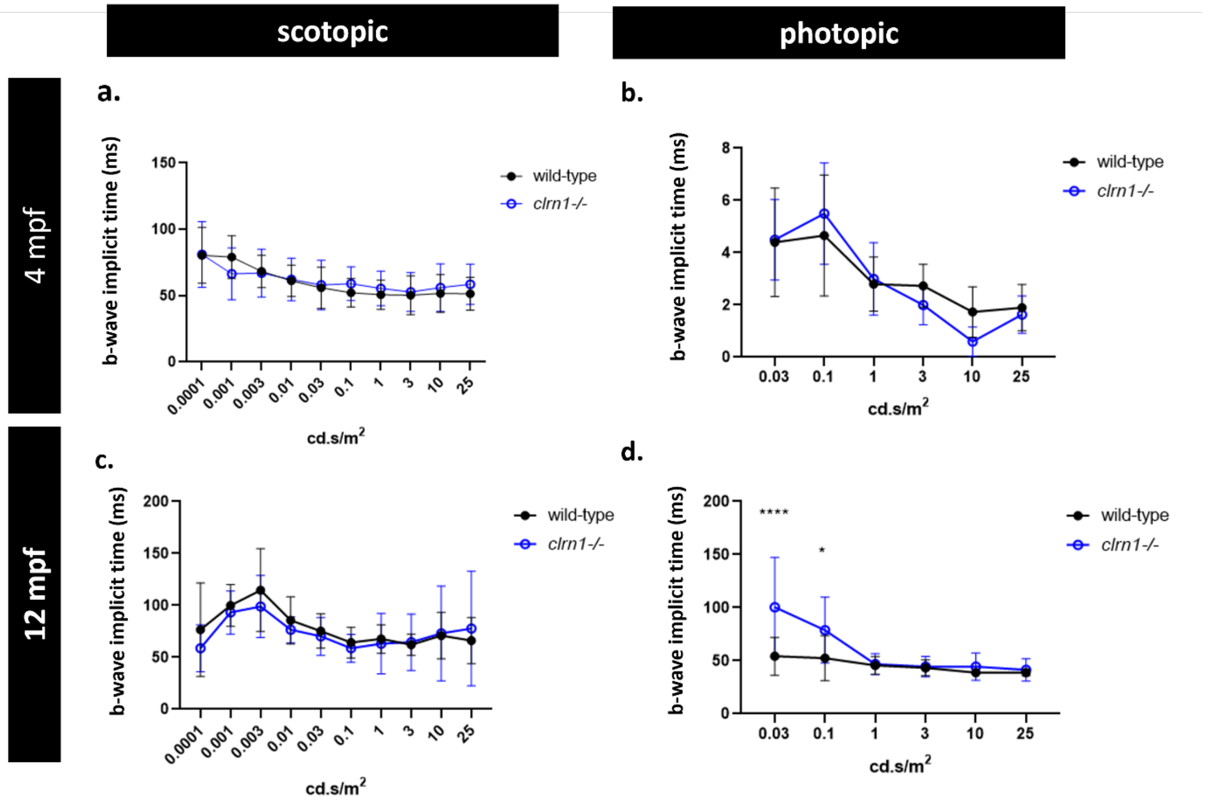

1036

1037 **Supplemental Figure 4 Scotopic and photopic B-wave implicit times at 4 and 12 mpf**

1038 Scotopic (a) and photopic (b) b-wave implicit time for wild-type and *clrn1*$^{-/-}$ zebrafish at 4 mpf.
1039 Scotopic (c) and photopic (d) b-wave implicit time for wild-type and *clrn1*$^{-/-}$ zebrafish at 12 mpf.
1040 (*p<0.05, ****p<0.001; One-way ANOVA) (n=10 per group). Error bars=SD.

1041

bioRxiv preprint doi: https://doi.org/10.1101/2024.02.29.582878; this version posted March 1, 2024. The copyright holder for this preprint (which was not certified by peer review) is the author/funder, who has granted bioRxiv a license to display the preprint in perpetuity. It is made available under aCC-BY 4.0 International license.

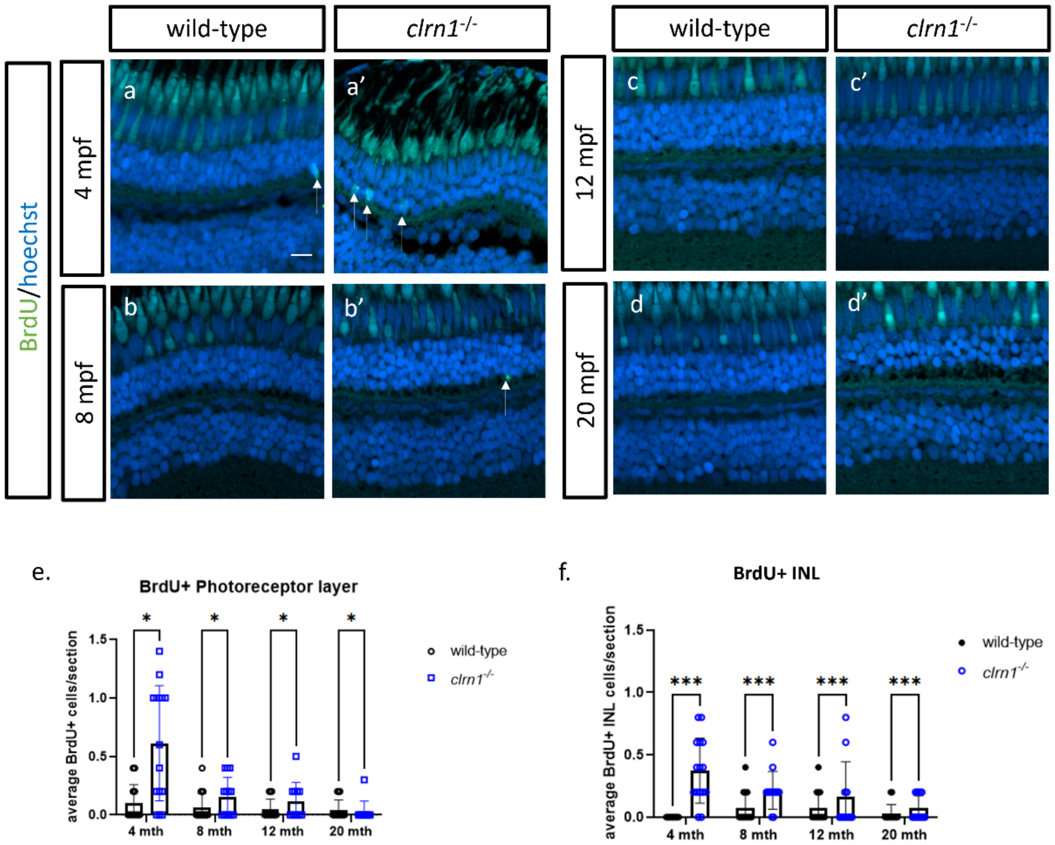

Supplemental Figure 5: BrdU incorporation is elevated in *clrn1$^{-/-}$* zebrafish at 4 mpf and decreases with age (a-d) Anti-BrdU (green) staining marks cells with BrdU incorporation, which serves as a measure of regeneration. Paraffin sections from 4 mpf (a), 8 mpf (b), 12 mpf (c), and 20 mpf (d) wild-type and *clrn1$^{-/-}$* zebrafish retinas. Quantification of Brdu+ nuclei in the photoreceptor (PR) (e) and inner nuclear layer (e) revealed an increase in BrdU incorporation for *clrn1$^{-/-}$* zebrafish at the youngest time point and this decreased with age. White arrows highlight BrdU+ nuclei. (**p<0.01; One-way ANOVA). Scale bar: 10 µm. White arrows denote Hoechst and BrdU positive nuclei.

bioRxiv preprint doi: https://doi.org/10.1101/2024.02.29.582878; this version posted March 1, 2024. The copyright holder for this preprint (which was not certified by peer review) is the author/funder, who has granted bioRxiv a license to display the preprint in perpetuity. It is made available under aCC-BY 4.0 International license.

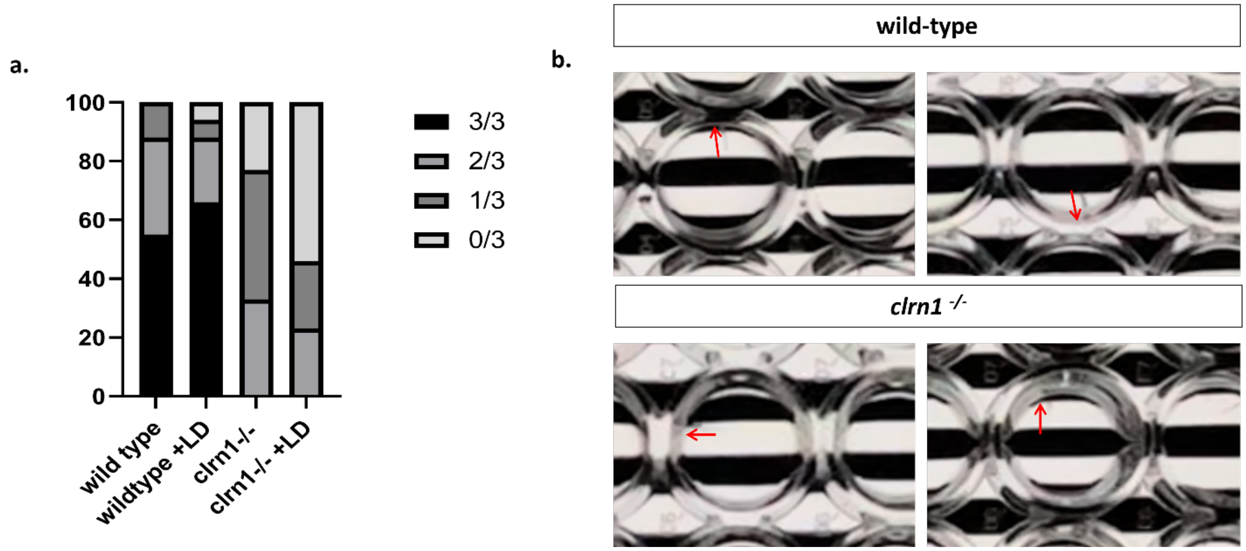

Supplemental Figure 6: OMR responses in wild-type and *clrn1*$^{-/-}$ zebrafish maintained in standard or high-intensity light conditions 5 to 7 dpf

Distribution of OMR responses in wild-type and *clrn1*$^{-/-}$ control or light damaged zebrafish (G) Representative examples of wild-type and *clrn1*$^{-/-}$ responses following stimulus direction change (H).

bioRxiv preprint doi: https://doi.org/10.1101/2024.02.29.582878; this version posted March 1, 2024. The copyright holder for this preprint (which was not certified by peer review) is the author/funder, who has granted bioRxiv a license to display the preprint in perpetuity. It is made available under aCC-BY 4.0 International license.

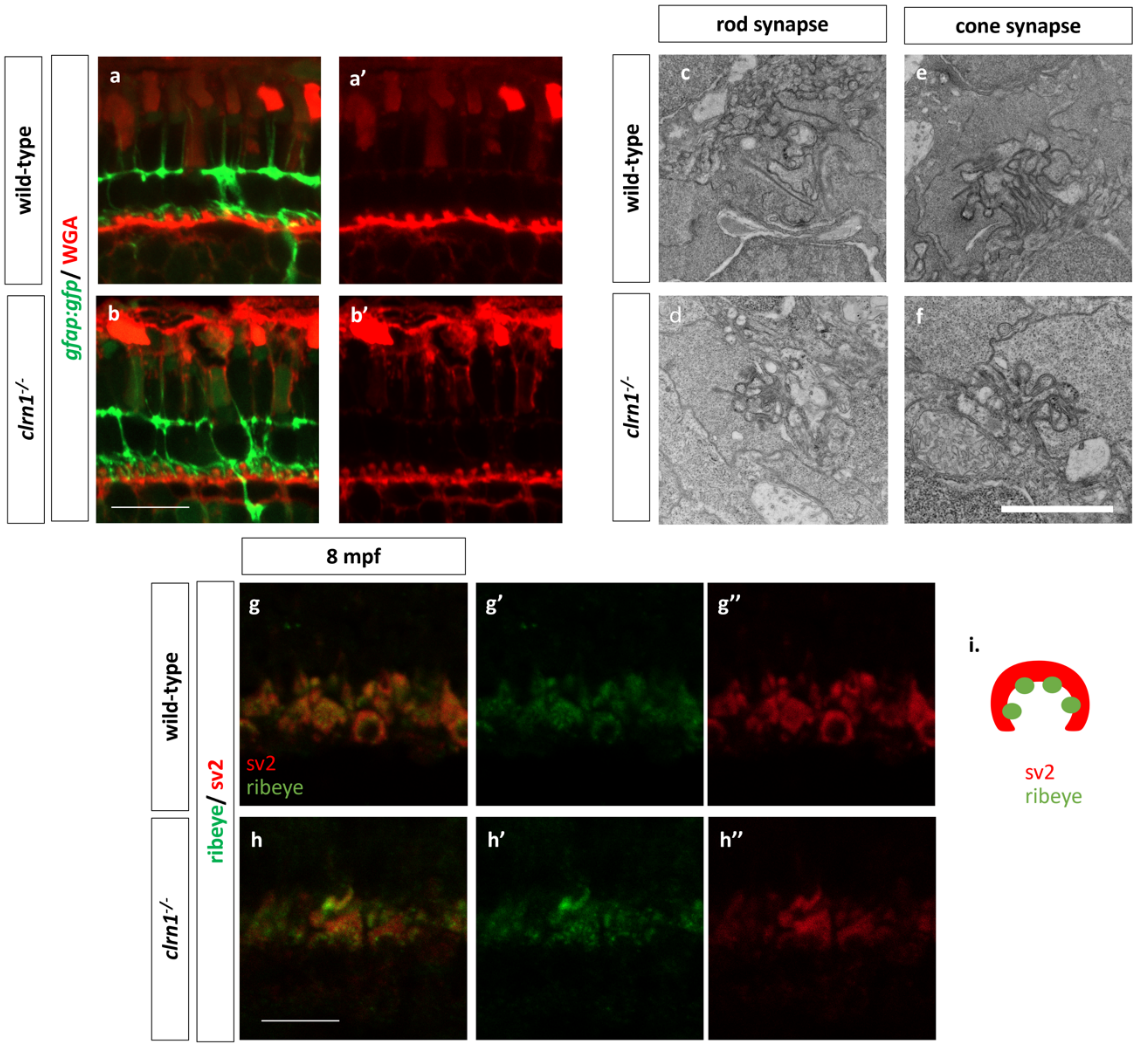

Supplemental Figure 7: Clrn1 is not required for synaptic development

(a, b) WGA (red) staining marks the lectin rich membrane of pre- and post-synaptic terminals, while Müller glia expressed GFP highlight cellular association with each synapse. There is no discernible difference between wild-type (a) and *clrn1*$^{-/-}$ zebrafish (b) at 7dpf. (c-f) Electron micrographs of rod and cone synapses in 7 dpf larvae. There were no discernable differences between wild-type rod (c) and cone (e) synapses compared to *clrn1*$^{-/-}$ rod (d) and cone (f) synapses at 7 dpf. (g-h) Anti-SV2 (red) and anti–ribeye (green) marks photoreceptor synapses. Analysis of protein localization and structure revealed no defects in synaptic vesicle localization and ribbon synapse formation in 8 mpf wild-type (g) and *clrn1*$^{-/-}$ zebrafish (h). (a-b, g-h) Scale bars: 10 µm, 1 µm (c-f), (Figure a-b, n=10 per group; Figure c-d n=3 per group, Figure g-h n=6-8 per group)

bioRxiv preprint doi: https://doi.org/10.1101/2024.02.29.582878; this version posted March 1, 2024. The copyright holder for this preprint (which was not certified by peer review) is the author/funder, who has granted bioRxiv a license to display the preprint in perpetuity. It is made available under aCC-BY 4.0 International license.

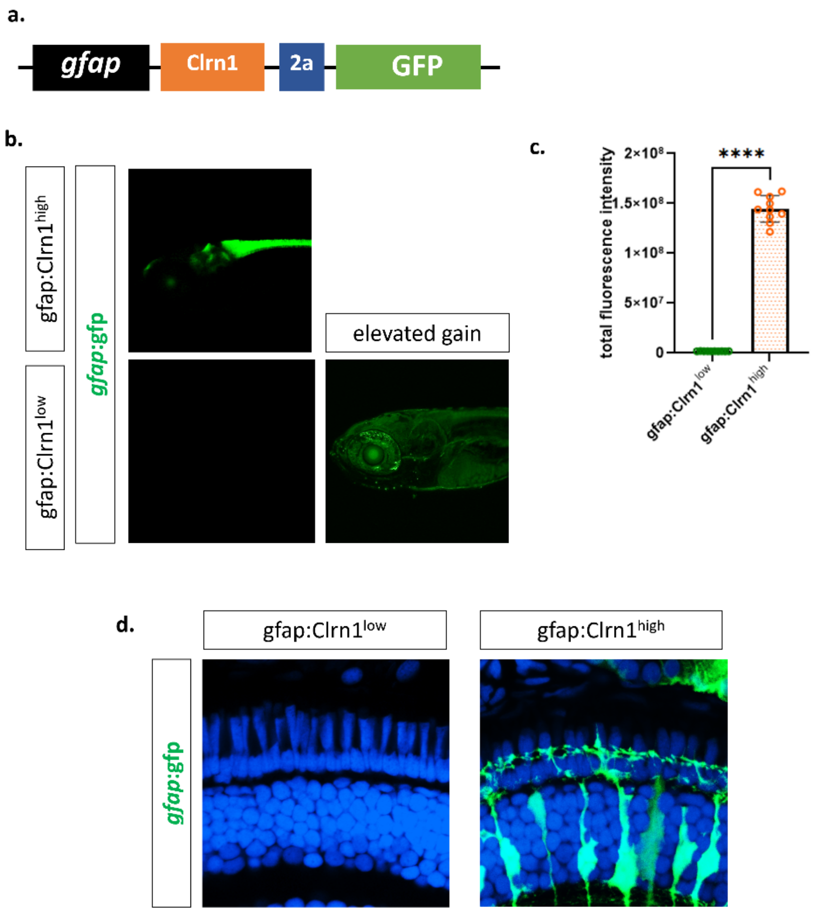

Supplemental Figure 8: Expression level difference between Clrn1 high and low re-expression in Müller glia transgenic lines

Representation of MG specific Clrn1 expression transgene (a) Representative image of the *Tg(gfap:Clrn1*^{low}*)* and *Tg(gfap:Clrn1*^{high}*)* and Representative image of *Tg(gfap:Clrn1*^{low}*)* at a higher gain to show reporter expression (b). Quantification of total GFP fluorescence intensity in the *Tg(gfap:Clrn1*^{high}*)* and *Tg(gfap:Clrn1*^{low}*)* (c). Transverse sections of 7dpf *Tg(gfap:Clrn1*^{low} and *Tg(gfap:Clrn1*^{high}*)* highlighting the loss of GFP signal in *Tg(gfap:Clrn1*^{low}*)* zebrafish tissue post fixation and processing (****$p<0.001$; Unpaired Students T-test). (n=10)

bioRxiv preprint doi: https://doi.org/10.1101/2024.02.29.582878; this version posted March 1, 2024. The copyright holder for this preprint (which was not certified by peer review) is the author/funder, who has granted bioRxiv a license to display the preprint in perpetuity. It is made available under aCC-BY 4.0 International license.

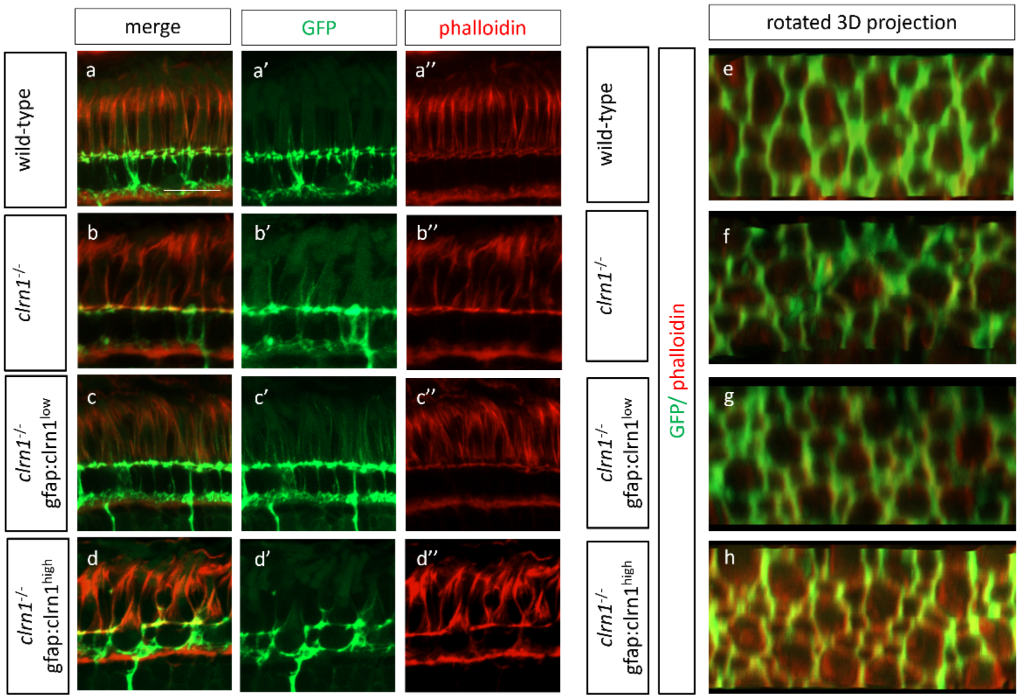

Supplemental Figure 9: Expression level of clrn1 in Müller glia affects the structure of Müller glia at the OLM and projections of apical microvilli

Comparison of Müller glia structure and actin staining along photoreceptor outersegments (a-d) and at the OLM (e-h) in wild-type (a, e), *clrn1*$^{-/-}$ (b, f), *clrn1*$^{-/-}$ *Tg(gfap:Clrn1*^{low}*)* (c, g), and *clrn1*$^{-/-}$ *Tg(gfap:Clrn1*^{low}*)* (d, f). To create images of the OLM, 3D projections from Z-stacks were generated and rotated in ImageJ. Comparison of Müller glia structure reveals that re-expression Clrn1 in *Tg(gfap:Clrn1*^{low}*) clrn1*$^{-/-}$ zebrafish corrects apical microvilli projects in Müller glia while higher re-expression significantly alters Müller glia structures and affects actin stain along outersegments. Scale Bar: 10 µm (Figure a-b, n=10-15 per group, Figure c-d n=3 per group)

bioRxiv preprint doi: https://doi.org/10.1101/2024.02.29.582878; this version posted March 1, 2024. The copyright holder for this preprint (which was not certified by peer review) is the author/funder, who has granted bioRxiv a license to display the preprint in perpetuity. It is made available under aCC-BY 4.0 International license.

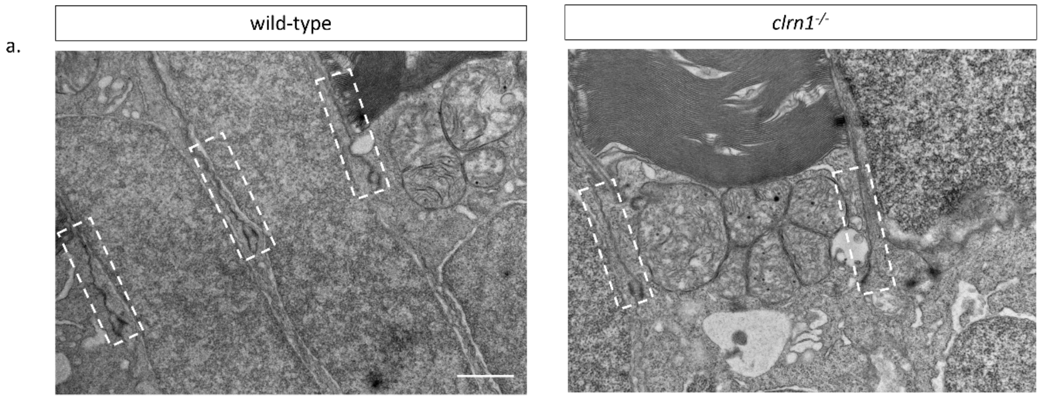

Supplemental Figure 10 Analysis of photoreceptor junction by TEM Representative images of the photoreceptor junctions for wild-type and *clrn1*$^{-/-}$ at 7dpf. White Boxes highlight junctions in the OLM Scale Bar: 1 uM

bioRxiv preprint doi: https://doi.org/10.1101/2024.02.29.582878; this version posted March 1, 2024. The copyright holder for this preprint (which was not certified by peer review) is the author/funder, who has granted bioRxiv a license to display the preprint in perpetuity. It is made available under aCC-BY 4.0 International license.

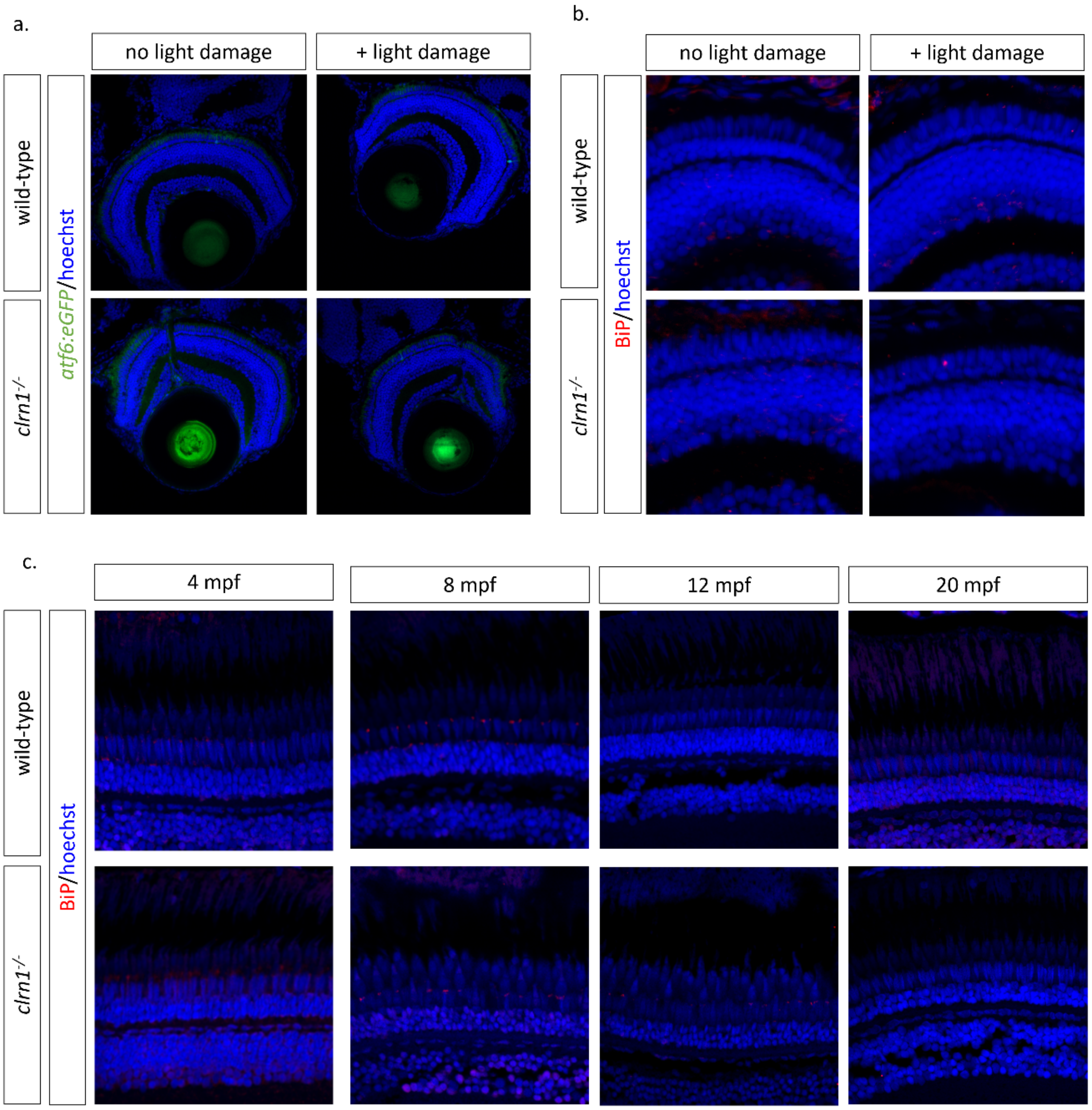

1223

1224 **Supplemental Figure 11: Markers of ER stress pathway activation are not elevated with**
1225 **the loss of Clrn1**

1226 Analysis of *Tg(atf6:eGFP)* reporter activation (a) and BiP (b) staining in 6 dpf larvae with or
1227 without 24 hours of light damage on transverse cryosections (a). BiP staining on 4-, 8-, 12-, and
1228 20-mpf wild-type and *clrn1*$^{-/-}$ zebrafish (c.)
